# Clinical Classification and Prognosis of Isolated Right-Sided Infective Endocarditis

**DOI:** 10.1097/MD.0000000000000137

**Published:** 2014-12-12

**Authors:** Carlos Ortiz, Javier López, Héctor García, Teresa Sevilla, Ana Revilla, Isidre Vilacosta, Cristina Sarriá, Carmen Olmos, Carlos Ferrera, Pablo Elpidio García, Carmen Sáez, Itziar Gómez, José Alberto San Román

**Affiliations:** From the Instituto de Ciencias del Corazón (ICICOR), Hospital Clínico Universitario, Valladolid (CO, JL, HG, TS, AR, PEG, IG, JASR); Hospital Clínico Universitario San Carlos (IV, CO, CF); and Servicio de Medicina Interna-Infecciosas, Instituto de Investigación del Hospital La Princesa, Madrid, Spain (CS, CS).

## Abstract

From an epidemiologic point of view, right-sided infective endocarditis (RSIE) affects different types of patients: intravenous drug users (IDUs), cardiac device carriers (pacemakers and implantable automatic defibrillators), and the “3 noes” endocarditis group: no left-sided, no IDUs, no cardiac devices. Our objective is to describe and compare the clinical profile and outcome of these groups of patients.

Every episode of infective endocarditis (IE) consecutively diagnosed in 3 tertiary centers from 1996 to 2012 was included in an ongoing multipurpose database. We assessed 85 epidemiologic, clinical, echocardiographic, and outcome variables in patients with isolated RSIE. A bivariated comparative analysis between the 3 groups was conducted.

Among 866 IE episodes, 121 were classified as isolated RSIE (14%): 36 IDUs (30%), 65 cardiac device carriers (54%), and 20 “3 noes” group (16%). IDUs were mainly young men (36 ± 7 years) without previous heart disease, few comorbidities, and frequent previous endocarditis episodes (28%). Human immunodeficiency virus infection was frequent (69%). Cardiac device carriers were older (66 ± 15 years) and had less comorbidities (34%). Removal of the infected device was performed in 91% of the patients without any death. The “3 noes” endocarditis group was composed mainly by middle-age men (56 ± 18 years), health care related infections (50%), and had many comorbidities (75%). Whereas *Staphylococcus aureus* were the most frequent cause in IDUs (72% vs 34% in device carriers and 34% in the “3 noes” group, *P* = 0.001), coagulase negative *Staphylococci* predominated in the device carriers (58% vs 11% in drug users and 35% in the “3 noes”, *P* < 0.001). Significant differences in mortality were found (17% in drug users, 3% in device carriers, and 30% in the “3 noes” group; *P* < 0.001).

These results suggest that RSIE should be separated into 3 groups (IDUs, cardiac device carriers, and the “3 noes”) and considered as independent entities as there are relevant epidemiologic, clinical, microbiological, echocardiographic, and prognostic differences among them.

## INTRODUCTION

Right-sided infective endocarditis (RSIE) represents 5% to 10% of all infective endocarditis (IE) episodes in adults.^1^ It can be divided into 3 groups according to the type of patient that host the disease: intravenous drug users (IDUs), cardiac device carriers (pacemakers and implantable automatic defibrillators), and the “3 noes” endocarditis group: no left-sided, no IDUs, and no cardiac devices. Classically, IDUs were responsible for most of the RSIE episodes, but this situation has changed in the last years because of an increase in cardiac device implantations.^2,3^ Therefore, numerous studies in the literature have described the features of RSIE in IDUs ^4–7^ and in cardiac device carriers, ^8–15^ but few have analyzed the “3 noes” group.^16–18^

Given that the patient profile is very different, we hypothesized that relevant differences exist among these groups regarding their profile and prognosis. If this assumption is corroborated, these 3 types of RSIE should be considered as separate entities. The objective of this study is to describe and compare the clinical, microbiologic, echocardiographic profile, and the outcome among RSIE in IDUs, cardiac device carriers, and the “3 noes” endocarditis group.

## METHODS

The study was conducted in 3 tertiary centers with cardiac surgery facilities, using standardized protocols, uniform data collection and identical diagnosis, and therapeutic criteria throughout the study, which have been previously described.^19^ Diagnosis of IE was made according to the Duke criteria until 2000^20^ and with the modified Duke criteria afterward.^21^ All patients underwent at least 1 transthoracic and transesophageal echocardiography.^22^ Once diagnosed, an initial empirical antibiotic treatment was initiated and then it was modified according to blood-culture results following the recommendations of the European guidelines on IE.^1,23^ Blood cultures were repeated within 48 to 72 hours from the initiation of the antibiotics to monitor the response.^24^ Systemic embolisms were diagnosed by imaging techniques such as computed tomography, magnetic resonance, and echography depending on their anatomic location. When pulmonary embolisms were clinically suspected, they were diagnosed by pulmonary computed tomography angiography or ventilation perfusion scintigraphy. During hospital admission, 85 variables were collected and grouped as follows: epidemiological, clinical, analytic, microbiological, echocardiographical, and outcome related. A comparative analysis was conducted. The study was approved by the ethical committees of our institutions.

### Definition of Terms

RSIE was considered nosocomial if the onset of symptoms had occurred >48 hours after admission. Heart failure was diagnosed by a clinical cardiologist according to the Framingham criteria.^25^ Chronic renal failure was defined as the presence of a glomerular filtration rate <60 mL/kg/min within the last 3 months and significant chronic anemia as serum hemoglobin levels <9 g/dL for at least 1 year. Uncontrolled infection was defined as persistent fever and positive blood cultures after 7 to 10 days with correct antibiotic treatment once excluded other potentially causes of fever.^1^ Septic shock was defined as the presence of sepsis, hypotension (<90 mm Hg), and organ perfusion abnormalities (oliguria, lactic acidosis, or altered mental status) that requires abundant fluid reposition and even pressor therapy.^26^Indications for surgery were made by a multidisciplinary group of cardiologists, cardiac surgeons, experts on infectious diseases, and microbiologists. In the cardiac device carriers group, for the purpose of analysis, we considered device extraction as cardiac surgery regardless of whether it was extracted with percutaneous traction or cardiac surgery. There were patients that despite having surgical indication did not underwent cardiac surgery because of high risk, patient's rejection, or death before surgery.

### Statistical Analysis

Continuous variables were expressed as mean ± standard deviation or median and interquartile rank. Categorical variables were expressed as absolute values and percentages. Normal distribution of numeric variables was verified with Kolmogorov–Smirnov test. Univariate analyses were conducted using the chi-square and the Fisher exact tests for categorical variables, whereas continuous variables were compared using analysis of variance or its nonparametric equivalent, Kruskal–Wallis test. All data were analyzed using version 20.0 of the Statistical Package for Social Sciences (SPSS Inc., Chicago, IL). For all analysis, a two-tailed *P* value of <0.05 was used to define statistical significance.

## RESULTS

**FIGURE 1:**
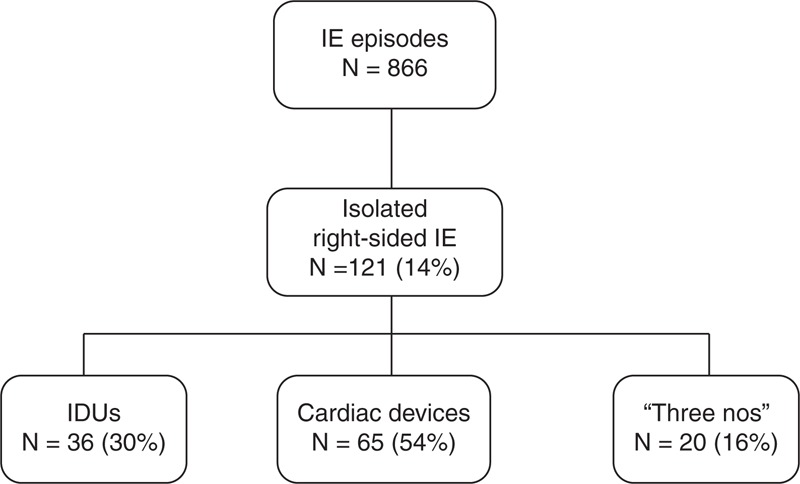
Classification of the patients with isolated RSIE. IDUs = intravenous drug users, IE = infective endocarditis, RSIE = right-sided infective endocarditis.

From 1996 to 2012, 866 IE episodes were consecutively diagnosed and included in an on-going database, which included 135 affected right-sided valves. Of those, 14 episodes were excluded from the analysis because of concomitant left valve infection. Our final study population is made up of 121 episodes of isolated RSIE in 115 patients (Figure 1): 36 episodes in IDUs (30%), 65 in cardiac device – 89% pacemaker, 11% implantable automatic defibrillator – carriers (54%), and 20 in the “3 noes” endocarditis group (16%). All patients were followed up during hospital admission regardless of the group they belonged to.

Relevant epidemiological changes have occurred in our series (Figure 1). Nowadays, RSIE affects predominantly cardiac device carriers representing two thirds of RSIE, whereas there has been a steady decline of RSIE in IDUs.

### Epidemiological Characteristics

**FIGURE 2:**
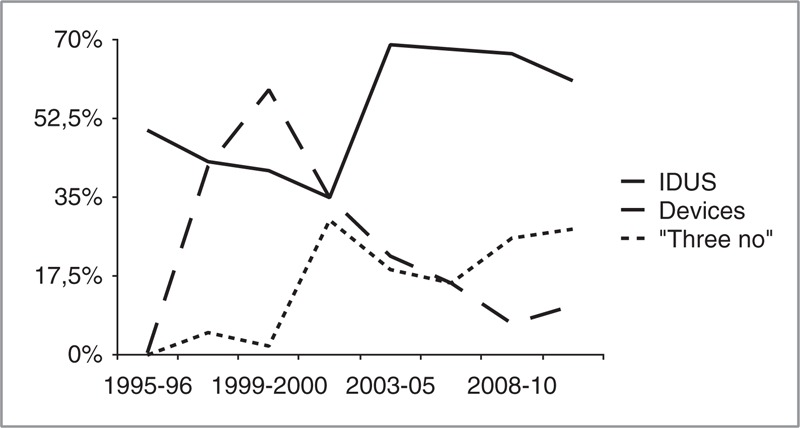
Epidemiological trends of isolated RSIE. IDUs = intravenous drug users, RSIE = right-sided infective endocarditis.

IDUs patients were the youngest and patients with cardiac devices the oldest. Seventy-six percent of RSIE were community acquired, especially in the IDUs group (97%), whereas nosocomial-related episodes were more frequent in the “3 noes” endocarditis group (50%) (Table 1; Figure 2).

**TABLE 1:**
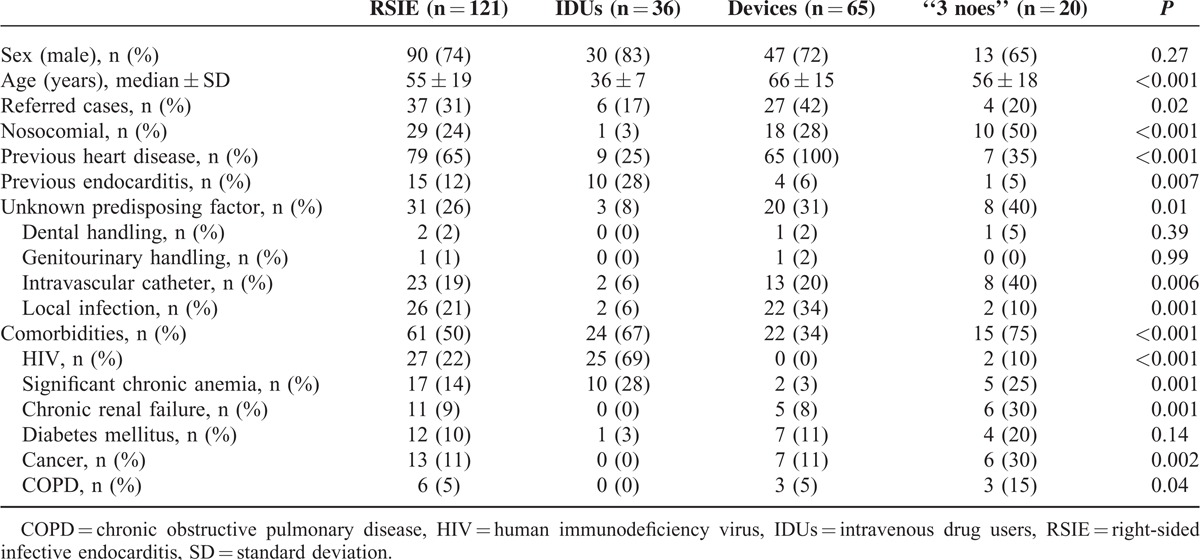
Comparison of Epidemiological Variables

The frequency of comorbidities and predisposing factors were significantly different in the 3 groups. Previous episodes of IE, human immunodeficiency virus (HIV) infection, and significant chronic anemia were more frequent in IDUs. Patients with devices had higher frequency of previous known heart disease and local infection. In the “3 noes” group, 40% of the episodes had no predisposing event and in other 40% an indwelling intravascular catheter was present. Comorbidities in this group were common, particularly significant chronic anemia, chronic renal failure (25% on dialysis), diabetes mellitus, chronic obstructive pulmonary disease (COPD), and cancer were the more frequently associated.

### Microbiological Characteristics

There were no differences in positive blood cultures at admission and within 48 to 72 hours from the initiation of the antibiotic treatment. *Staphylococcus aureus* (45%) and coagulase-negative *Staphylococci* (40%) were the most frequently isolated microorganisms overall. *Staphylococcus aureus* was the leading cause in IDUs, whereas coagulase-negative *Staphylococci* prevailed in patients with devices. In the “3 noes” group both bacteria had the same frequency (35%). Gram-negative bacilli were responsible for 10% of the episodes in the “3 noes” group. Only 1 case of fungal endocarditis in IDUs was observed. Overall, 12 episodes were polymicrobial (3 in IDUs, 7 in devices, and 2 in “3 noes”) (Table 2).

**TABLE 2:**
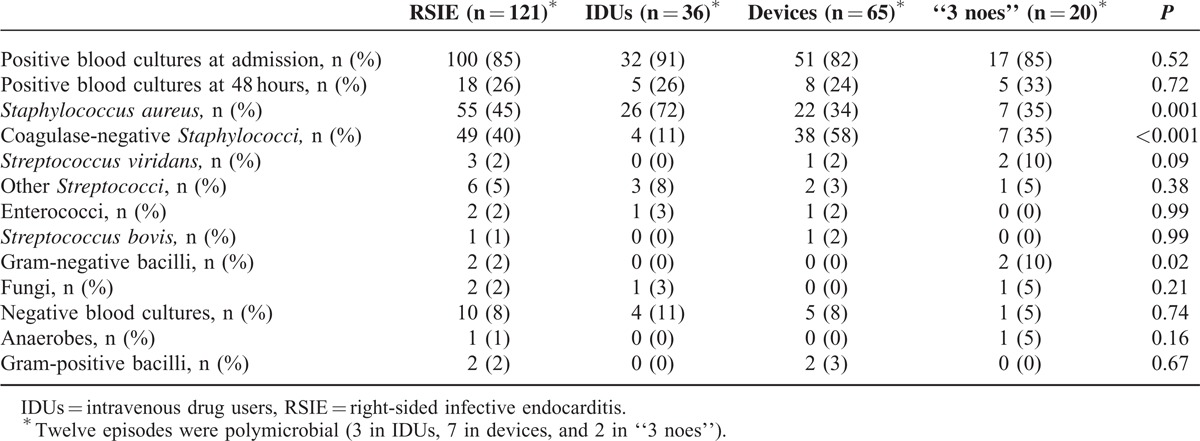
Comparison of Microbiological Variables

### Clinical, Echocardiographic, and Outcome Characteristics

IDUs patients had a high incidence of pulmonary embolisms (53%). Patients in the “3 noes” group had more renal failure (30%), septic shock (15%) and uncontrolled infection (55%) than the other groups (Table 3).

**TABLE 3:**
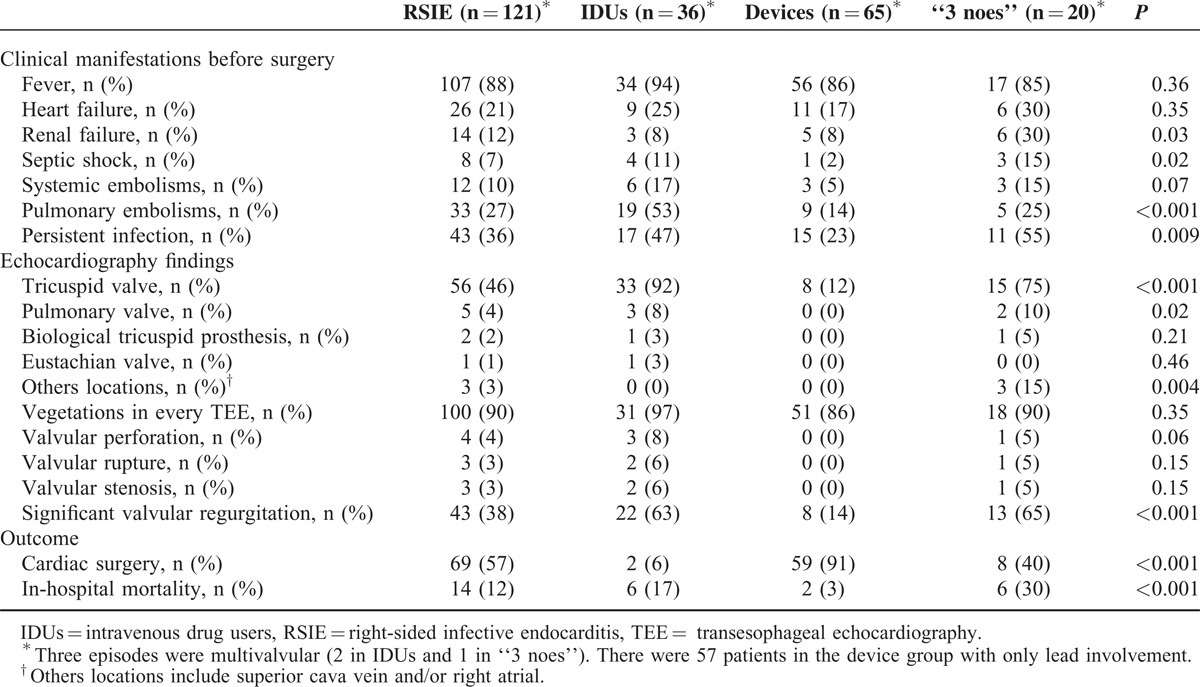
Comparison of Clinical, Echocardiographic, and Outcome Variables

Regarding echocardiographic findings, the tricuspid valve was the most frequently affected in the 3 groups, but only 11% of the patients in the cardiac devices group had valvular involvement. Significant valvular regurgitation was present in 63% of IDUs and 65% in the “3 noes” group. No other echocardiographic differences were found. Overall, 3 episodes were multivalvular (2 in IDUs and 1 in “3 noes”).

Relevant differences were observed in respect to the therapeutic approach and short-term prognosis: device and leads removal was performed in 91% of patients with devices, with very low in-hospital mortality (3%). The 2 deaths occurred in patients in whom extraction of the infected device was not performed because their clinical situation was too critical to consider any kind of invasive procedure. Surgery was rarely performed in IDUs and mortality was 17%. The “3 noes” group had the highest mortality (30%), despite a higher use of surgery (40%).

## DISCUSSION

Our study demonstrates significant epidemiologic, clinical, microbiological, echocardiographic, and prognostic differences among RSIE in IDUs, cardiac devices carriers, and the “3 noes” patients, providing evidence to support the consideration as separate entities.

Unlike other series of RSIE that have included patients with concomitant left-sided involvement,^6–7,12,14^ we decided to exclude these patients from our analysis, as their findings might be otherwise biased. This may explain some of the differences between our results and those of other previously published series.

### IE in IDUs

Intravenous drug addiction involves multiple associated medical problems, infections being the most frequently related to hospital admissions and mortality.^26^ Classically, drug use was considered one of the most important risk factors for the development of IE in developed countries.^27,28^

In our series, IDUs patients are mainly young men without previous heart disease and almost one third have had at least 1 previous episode of IE in the past. This finding is shared with other series^29^ and can be related to the nonstop drug addiction consumption of many of these patients. The only frequent comorbidity is the HIV infection, affecting almost 3 quarters of patients.

Causative microorganisms more often isolated are *Staphylococcus aureus*, followed by coagulase-negative *Staphylococci*, most of them meticillin sensitive (93%), reflecting that these infections are community acquired. In contrast to previous series of RSIE in IDUs, where *Streptococcus viridans* were responsible of 25% of the episodes,^5,7^ we did not found any case. This might be related to the exclusion of patients with concomitant left-sided infection.

Tricuspid valve is the most frequently affected valve (92%) followed by the pulmonary valve (8%). Two episodes were multivalvular. The infection was poorly controlled in many patients, as almost 50% of patients have persistent infection. Septic shock was the main cause of death in this group. Their immunosuppressive state and the low percentage of patients who underwent cardiac surgery might have altered infection control and justify the high mortality when compared with other series.^5,7^

### IE in Cardiac Device Carriers

In our series, IE in cardiac devices carriers accounted for more than half of RSIE episodes. Moreover, a steady increase in the incidence or this disease is expected within the next years because of the spread indications of device implantation, mainly as primary prevention^2^ and the higher life expectancy after the implantation with the consequent need of reoperation for battery end of life or lead dysfunction. They are associated with important healthcare costs^2^ and high mortality rates (ranging from 7% to 17%).^8,10–14,30^

Pocket infections, the so-called local device infections, and the presence of intravascular catheters are the most common predisposing conditions in our series. Other described risk factors include the use of temporary pacemakers, early reoperation (before hospital discharge) for hematoma or lead replacement,^9^ prolonged use of corticosteroids and the presence of >2 leads.^11^ The use of prophylactic antibiotics before implantation has been reported as a protective factor.^9,11^

As expected, the microorganisms most frequently associated in our series were coagulase-negative *Staphylococci*, followed by *Staphylococcus aureus*.^12,14,30^ Consistent with other series,^30^ a high rate of episodes caused by meticillin-resistant *Staphylococci* was observed (30%). Thus, antibiotics active against meticillin-resistant *Staphylococci* spp. should be strongly considered as part of the antibiotic prophylaxis before device implantation.

Management of these patients is based in two important points: complete device removal and intravenous antibiotics. Most of our patients (91%) underwent urgent device extraction by means of percutaneous traction or cardiac surgery with very low incidence of extraction-related complications and without any death. After device extraction, we completed 2 to 4 weeks of antibiotics guided by antibiogram. In-hospital mortality in our series was 3% and the only 2 deaths occurred in patients with a very deteriorated clinical status in whom invasive procedures were not considered. In-hospital mortality in our series is lower than previously reported by others groups (11%–17%),^8,30^ which can be explained not only by the high rate of device removal, but also by the fact that we have only included patients with isolated RSIE. Therefore, our results reinforce the need of removal of the infected device in these patients as soon as IE is diagnosed.

### The “3 Noes” Endocarditis Group

Little evidence is available regarding the “3 noes” endocarditis group. The majority of articles published on this topic were case studies or retrospective compilation of cases.^16,17,31–39^ There is only 1 prospective series of this population.^18^

From an epidemiological point of view, the average profile of this group is represented by middle-age men, with frequent comorbidities such as renal failure, dialysis, diabetes mellitus, cancer, and COPD. Some of these patients usually have the presence of intravascular catheters, which are known as the main source of bacteremia, particularly by *Staphylococci*.^40^

With regards to microbiology, some aspects merit discussion. First, although the most common causative microorganism are *Staphylococcus* spp., the frequency is much lower than in the other 2 groups, and *Streptococcus* spp. increase in importance. Second, meticillin-resistant *Staphylococcus* spp. is frequent (33%), which suggests health care related cause.

As described previously, a characteristic finding in these patients is the “tricuspid syndrome”, which consists of respiratory events (pneumonia, pulmonary embolisms), anemia, and microscopic hematuria.^17^ It is present in 28% of our “3 noes” endocarditis patients.

From a practical point of view, we suggest that in patients with persistent fever and respiratory signs and symptoms, RSIE should be kept in mind in the differential diagnosis, even though classical predisposing factors (cardiac devices and intravenous drug use) may not be present.

The most important difference from the other 2 groups is that in-hospital mortality in this group is high, with rates similar to left-sided IE (30%). This high mortality rate might be explained not only by a diagnosis delay, but also by the fact that these patients presented more comorbidities, worse clinical features at admission (renal failure and septic shock), a high prevalence of meticilin-resistant *Staphylococci*, and a high rate of persistent infection. Furthermore, interpretation of data should be performed with caution given the small sample size.

## LIMITATIONS

We are aware of several limitations of our work. Some episodes of RSIE in patients admitted to our hospitals may have not been diagnosed for 2 reasons: first, we used the Duke and the modified Duke criteria for the diagnosis of RSIE, which are considered inappropriate for this condition by some authors.^41,42^ Second, as the clinical manifestations and radiographic findings are similar to a much more common disease, such as respiratory infection, many of these patients were treated with antibiotics and had a good clinical course, hence, the diagnosis of RSIE may have been missed. Another limitation is that the patients in our series came from tertiary centers, with the consequent selection bias inherent to this type of hospital. These limitations should be taken into account when interpreting the results. Finally, due to the low number of events (only 14 deaths), we could not perform a multivariate analysis to test the association between having a poor outcome and the type of episode.

## CONCLUSION

These results suggest that RSIE may be classified into 3 groups (IDUs, cardiac device carriers, and the “3 noes”), which can be considered as independent entities because of the relevant epidemiologic, clinical, microbiological, echocardiographic, and prognostic differences among them. The low in-hospital mortality in cardiac device carriers reinforces the need to remove the infected device in these patients as soon as IE is diagnosed.
